# Flow Separation and Turbulence in Jet Pumps for Thermoacoustic Applications

**DOI:** 10.1007/s10494-016-9731-8

**Published:** 2016-05-23

**Authors:** Joris P. Oosterhuis, Anton A. Verbeek, Simon Bühler, Douglas Wilcox, Theo H. van der Meer

**Affiliations:** 1 Department of Thermal Engineering, University of Twente, Enschede, The Netherlands; 2 Chart Inc., Troy, NY USA

**Keywords:** Jet pumps, Thermoacoustics, Flow separation, Turbulence, Oscillatory flows, Minor losses

## Abstract

The effect of flow separation and turbulence on the performance of a jet pump in oscillatory flows is investigated. A jet pump is a static device whose shape induces asymmetric hydrodynamic end effects when placed in an oscillatory flow. This will result in a time-averaged pressure drop which can be used to suppress acoustic streaming in closed-loop thermoacoustic devices. An experimental setup is used to measure the time-averaged pressure drop as well as the acoustic power dissipation across two different jet pump geometries in a pure oscillatory flow. The results are compared against published numerical results where flow separation was found to have a negative effect on the jet pump performance in a laminar flow. Using hot-wire anemometry the onset of flow separation is determined experimentally and the applicability of a critical Reynolds number for oscillatory pipe flows is confirmed for jet pump applications. It is found that turbulence can lead to a reduction of flow separation and hence, to an improvement in jet pump performance compared to laminar oscillatory flows.

## Introduction

Thermoacoustic engines are an interesting alternative to conventional heat engines due to the lack of moving parts in the hot region and the low temperature difference required to operate. These engines provide a durable solution in, for example, waste heat recovery applications [1]. With traveling wave based thermoacoustic devices, which consist of a closed-loop tube, high theoretical efficiencies can be achieved [2]. Backhaus & Swift applied the traveling wave concept in their engine, reaching a thermal–to–acoustic efficiency of 30 % [3].

**Fig. 1:**
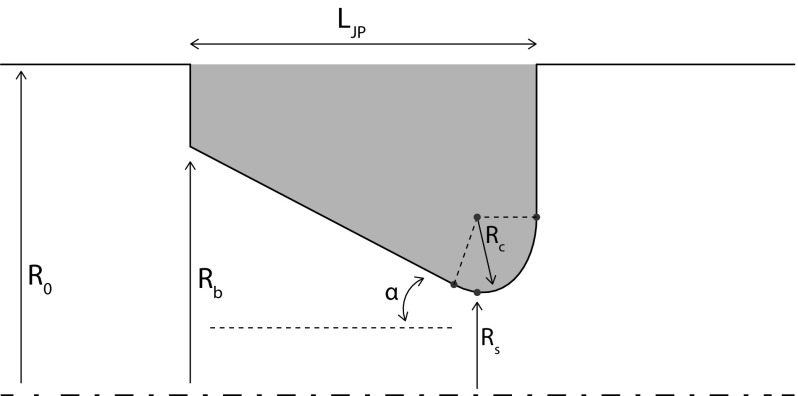
Schematic of jet pump geometry with dimensions (not to scale). Bottom line indicates centerline, top solid line indicates outer tube wall. Reproduced with permission from [9]. Copyright 2015, Acoustical Society of America

Despite the higher efficiency, the traveling wave configuration has one major disadvantage: due to the closed looped geometry a time-averaged mass flow, known as “Gedeon streaming”, can occur [4]. This type of acoustic streaming leads to undesired convective heat transport, which reduces the efficiency of closed-loop thermoacoustic devices. A commonly used solution to avoid Gedeon streaming is the application of a jet pump [3, 5–7]. A jet pump is a section with a tapered hole as depicted in Fig. 1. The combination of an oscillatory flow and an asymmetry in the hydrodynamic end effects results in a time-averaged pressure drop across the jet pump. By balancing this time-averaged pressure drop with the pressure drop that exists across the regenerator of the thermoacoustic device, Gedeon streaming can be suppressed [3].

The current design methodology for jet pumps is based on a quasi-steady approximation [3]. Using minor loss coefficients reported for the abrupt expansion and contraction in steady pipe flow, the time-averaged pressure drop and related acoustic power dissipation in an oscillatory flow can be estimated,

1 $$\begin{array}{@{}rcl@{}} \Delta p_{2} &=& \frac{1}{8}\rho_{0} |u_{1,JP}|^{2}\left[(K_{exp,s}-K_{con,s}) + \left( \frac{A_{s}}{A_{b}}\right)^{2}(K_{con,b}-K_{exp,b})\right], \end{array} $$

2 $$\begin{array}{@{}rcl@{}} \Delta \dot{E}_{2} & =& \frac{\rho_{0} |u_{\mathit{1},JP}|^{3}A_{s}}{3\pi}\left[(K_{exp,s}+K_{con,s}) + \left( \frac{A_{s}}{A_{b}}\right)^{2}(K_{con,b}+K_{exp,b})\right], \end{array} $$

with *K*_*con*_ and *K*_*exp*_ representing minor loss coefficients for contraction and expansion, respectively. The subscripts “*s*” and “*b*” indicate the small and big opening area of the jet pump hole (see Fig. 1). *u*_*1*,*JP*_ is the cross-sectional averaged velocity amplitude in the jet pump small opening, referred to as the jet pump “waist”. An optimal jet pump design should generate the required Δ*p*_2_ to cancel Gedeon streaming in the thermoacoustic device while minimizing the jet pump’s associated acoustic power dissipation.

**Fig. 2:**
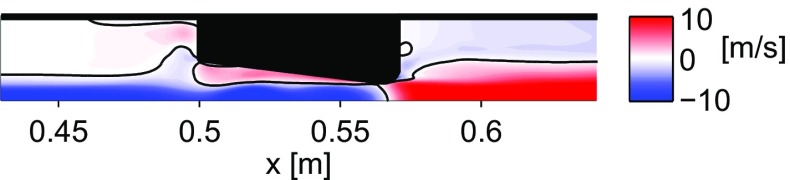
Simulated time-averaged axial velocity field using a 7 ^∘^ taper angle jet pump driven at 100 Hz, *K* *C*_*α*_=0.72. Reproduced with permission from [9]. Copyright 2015, Acoustical Society of America

Petculescu & Wilen showed that the jet pump taper angle, whose effect is not included in the quasi-steady approximation, has a significant effect on a jet pump’s performance [8]. In a recent numerical study we have determined four different flow regimes as a function of the jet pump geometry and wave amplitude and we have shown that the applicability of the quasi-steady approximation in laminar oscillatory flows is limited [9]. Due to the taper of the jet pump’s through-hole, the flow in the leftward direction of Fig. 1 can separate from the jet pump wall, resulting in a rightward time-averaged velocity close to the jet pump wall and a leftward time-averaged velocity at the centerline. The latter is visible in Fig. 2 where the simulated time-averaged velocity field is shown for a case where flow separation was observed. The flow separation leads to a significant decrease in the time-averaged pressure drop and to a large deviation from the quasi-steady approximation. The onset of flow separation coincides with vortices propagating through the jet pump and is found to be dependent on the Keulegan-Carpenter number, which is based on the diameter of the jet pump waist (*D*_*s*_=2⋅*R*_*s*_ in Fig. 1) and the jet pump taper angle *α* (in radians),

3 $$ KC_{\alpha} = \frac{\xi_{1}}{D_{s}}\alpha .  $$

Here, *ξ*_1_ is the acoustic particle displacement amplitude at the jet pump waist determined from the velocity amplitude and angular frequency, *ξ*_1_=|*u*_*1*,*JP*_|/*ω*. For a jet pump geometry with a “smooth” waist (*R*_*c*_/*D*_*s*_=0.36), clear flow separation was observed at *K* *C*_*α*_>0.7 and the jet pump performance was significantly reduced [10]. This is in line with the findings of King & Smith on oscillatory flow separation in a two-dimensional diffuser [11]. They observed that the higher the displacement amplitude, the earlier in the acoustic cycle the flow separates. This results in a larger time-averaged pressure drop in the diverging direction. An increase in the diffuser angle also results in flow separation occurring earlier in the acoustic cycle and larger minor losses. Furthermore, they found the acoustic Reynolds number to have an impact on the flow separation. Both the time-averaged pressure drop and acoustic power dissipation reduced with increasing Reynolds number which is an important motivation for the current investigation.

In order to design effective and robust jet pumps, it is important to predict the occurrence of flow separation due to its degrading effect on a jet pump’s performance. In the current article the influence of turbulence and flow separation in conical jet pumps is investigated experimentally in both laminar and turbulent oscillatory flows. After a description of the experimental setup in Section 2, the jet pump performance in terms of the time-averaged pressure drop and acoustic power dissipation is measured (Section 3). It will be shown that there exists a difference in jet pump performance between the laminar and turbulent regime. Subsequently, hot-wire anemometry is used to further characterize the turbulence and occurrence of flow separation in two different jet pump geometries (Sections 4–5).

## Experimental Setup

**Fig. 3:**
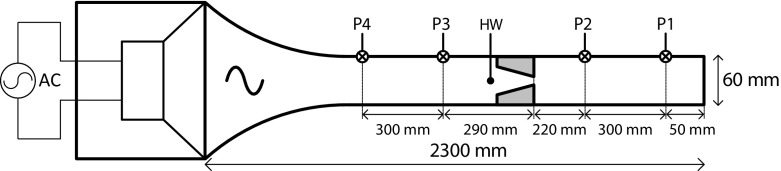
Schematic of the experimental setup with the pressure sensors (P1 – P4), hot-wire probe (HW) and jet pump sample. Dimensions not to scale

The experimental setup is shown schematically in Fig. 3 and is similar to the setup previously used by Aben [12]. On the left side, a loudspeaker (JBL W16GTi) is mounted with a cylindrical back volume; both are structurally decoupled from the rest of the setup by a membrane. A sinusoid signal generated by a computer sound card is amplified using a 2kW audio amplifier (Behringer EP2000). The acoustic wave propagates through a horn to the tube section which has an inner diameter of 60 mm and a total length of 1.2 m. The jet pump is mounted in a 400 mm long transparent PMMA section of the tube. Vibration of the jet pump samples with respect to the outer tube housing has been ruled out by using high-speed camera visualization at 1000 fps to determine the mutual displacements of the two parts. The setup is filled with air at ambient conditions. The effect of wave phasing (i.e., standing wave or traveling wave) on the jet pump performance has been investigated previously and no significant differences were observed [13]. To achieve the maximal acoustic amplitude with the current setup, a closed termination is used in all presented experiments.

### Pressure measurement system

In order to quantify the jet pump performance, four piezo-resistive differential pressure sensors (Honeywell 26PCAFA6D) are mounted flush with the tube wall. On either side of the jet pump two pressure sensors are located with a mutual distance of 300 mm (see Fig. 3). After amplification, the sensor signals are acquired using a NI-6250 data acquisition device at a sampling frequency of *f*_*s*_=20 kHz and a sampling time of *T*_*s*_=1 s. The pressure sensors are dynamically calibrated to a pre-calibrated Kulite XTE-190M pressure sensor in a frequency and pressure amplitude range of 20Hz to 150Hz and 100Pa to 2500Pa, respectively. The calibration setup consists of a closed tube with a loudspeaker (Monacor SP-60/8) at one end, which is used to generate an acoustic field. At the other side of the tube, the Kulite reference sensor and an uncalibrated Honeywell sensor are mounted flush with the end flange. This dynamic calibration procedure yields a typical averaged sensor sensitivity over the full calibration range of 1 mV/Pa The standard deviation in the sensitivity is less than 1 μV/Pa across the calibration range. The phase accuracy is determined in the same calibration procedure. A constant time delay is observed yielding a mutual phase difference between the four sensors of less than 0.32 ^∘^ at 100 Hz. This difference is taken into account as a measurement error. The linearity of the Honeywell pressure sensors is measured using a static water column calibration up to 2500 Pa. The maximum error due to non-linearity is ±1 *%* of reading for pressures up to 500 Pa and ±0.2 *%* of reading for *p*>500 Pa.

### Data analysis

After digitally phase-locking the acquired pressure signals, the pressure amplitude *p*_1_ is calculated from the discrete Fourier transform at the corresponding driving frequency. The time-averaged pressure *p*_2_ at each sensor is calculated by averaging the signal over an integer number of wave periods. This removes the contribution of the acoustic wave from the signal. The time-averaged pressure drop over a jet pump sample, Δ*p*_2_, is given by the difference in *p*_2_ from sensors 2 and 3 (see Fig. 3).

In order to determine the velocity amplitude in the jet pump waist *u*_*1*,*JP*_ in a non-invasive way, a two-dimensional linear acoustic model of the setup is employed. By relating the calculated velocity amplitude in the jet pump waist to the pressure amplitude at one of the sensor locations, a linear conversion factor between pressure and velocity is determined from the acoustic model. This conversion factor is only dependent on the driving frequency and the position of the jet pump in the setup. A comparison with non-linear, laminar CFD results confirmed that this approach is accurate to within 5 % when the pressure field to the right of the small jet pump opening is used as a reference (i.e., sensor 1 or 2 in Fig. 3).

Furthermore, the measured pressure amplitudes are used to calculate the acoustic power $\dot {E}_{2}$ on either side of the jet pump for which the method of Fusco et al. is used [14]. By taking the difference between the acoustic power on either side of the jet pump and correcting for dissipative effects in the tube segments, the contribution of the jet pump to the acoustic power dissipation $\Delta \dot {E}_{2}$ is found.

## Jet Pump Performance

Two jet pump samples are investigated, each having a different taper angle. The dimensions are identical to the geometries used in a previous numerical study [9] and are shown in Table 1. The samples are manufactured from a Nylon polymer (PA 2200) using a 3D laser sintering rapid prototyping process and polished. The surface roughness is measured and ranges from *R*_*A*_=8.5 μm to 12 μm.

**Table 1:** Dimensions of jet pump samples

Sample	*α*	*L*_*JP*_	*R*_*b*_	*R*_*s*_	*R*_*c*_
1	7 ^∘^	70.5 mm	15.0 mm	7.0 mm	5.0 mm
2	15 ^∘^	35.5 mm	15.0 mm	7.0 mm	5.0 mm

Nomenclature according to Fig. 1

Following the quasi-steady approximation (1–2), the time-averaged pressure drop across the jet pump is expected to scale with |*u*_*1*,*JP*_|^2^ while ${\Delta }\dot {E}_{2}$ scales with |*u*_*1*,*JP*_|^3^. As such, the measured time-averaged pressure drop Δ*p*_2_ and acoustic power dissipation ${\Delta }\dot {E}_{2}$ are normalized according to [9, 15]

4 $$\begin{array}{@{}rcl@{}} {\Delta} p_{2}^{*} &=& \frac{8{\Delta} p_{2}}{\rho_{0} |u_{1,JP}|^{2}}, \end{array} $$

5 $$\begin{array}{@{}rcl@{}} {\Delta} \dot{E}_{2}^{*} &=& \frac{3\pi{\Delta} \dot{E}_{2}}{\rho_{0}\pi R_{s}^{2} |u_{1,JP}|^{3}} , \end{array} $$

with ${\Delta } p_{2}^{*}$ representing the difference in minor loss coefficients between the backward and forward flow direction and ${\Delta }\dot {E}_{2}^{*}$ representing the summation of minor loss coefficients, assuming the quasi-steady approximation to be valid (1–2). By examining these normalized quantities, any effect flow separation and turbulence have on the jet pump performance becomes more readily visible.

**Fig. 4:**
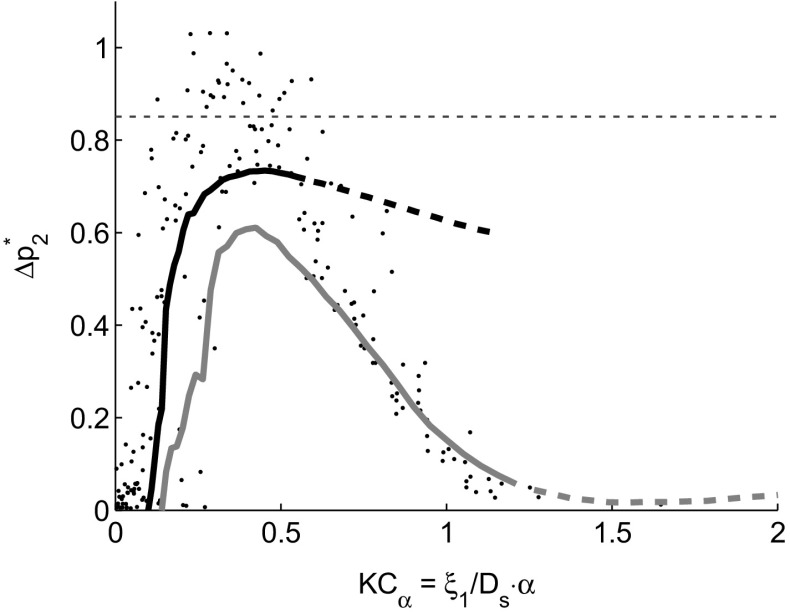
Dimensionless pressure drop measured experimentally at 80 Hz for two jet pump samples: 7 ^∘^ taper angle jet pump (black line) and 15 ^∘^ jet pump (gray line). Dots represent numerical results from jet pump geometries with taper angles ranging from 3 ^∘^ to 20 ^∘^ and driven at frequencies ranging from 10 Hz to 200 Hz reproduced from [10]. Experimental results where *R* *e*>*R* *e*_*c*_ are shown by a dashed line. The horizontal dashed line indicates the expected performance from the quasi-steady approximation [3]

For each jet pump sample, a sweep is executed over the wave amplitude by increasing the audio volume in 50 consecutive steps. At each setpoint, the pressure is recorded for 60 time traces of 1 s each, and the outcome variables (see Section 2) are subsequently averaged. Fig. 4 shows the dimensionless time-averaged pressure drop as a function of the Keulegan-Carpenter number *K* *C*_*α*_ defined in Eq. 3. The lines represent experimental results obtained at 80 Hz using the 7 ^∘^ taper angle jet pump (upper black line) and using the 15 ^∘^ taper angle jet pump (lower gray line). The dots represent published numerical results using a large variety of jet pump geometries with a taper angle ranging from 3 ^∘^ to 20 ^∘^ and simulated at frequencies ranging from 10 Hz to 200 Hz [10]. All the numerical results show an increase in ${\Delta } p_{2}^{*}$ at low values of *K* *C*_*α*_. This is a result of minor losses caused by the vortex shedding from the small jet pump opening. As soon as the flow starts to separate from the inside jet pump wall, the dimensionless time-averaged pressure drop stagnates, then drops rapidly when full flow separation without reattachment is observed for *K* *C*_*α*_>0.7. The experimental results (lines in Fig. 4) show a similar increase and maximum in ${\Delta } p_{2}^{*}$. However, the pressure drop tends to stabilize for higher values of *K* *C*_*α*_ and higher Reynolds numbers. This suggests a reduction of flow separation at high Reynolds numbers, especially in the case of the 7 ^∘^ jet pump sample. A major phenomenon that can explain the hypothesized reduction of flow separation is the occurrence of turbulence. Hence, we define an acoustic Reynolds number based on the viscous penetration depth $\delta _{\nu }=\sqrt {2\nu /\omega }$ with *ν* being the kinematic viscosity,

6 $$ Re = \frac{|u_{\mathit{1},JP}|\delta_{\nu}}{\nu}.  $$

Its critical value for oscillatory pipe flows is defined as [16],

7 $$ Re_{c} = 305\left( \frac{D}{\delta_{\nu}}\right)^{\frac{1}{7}},  $$

In Fig. 4, the dashed parts of the curves represent results where *R* *e*>*R* *e*_*c*_ and a transition to turbulence can be expected. It is remarkable that in the turbulent regime, little additional decay in ${\Delta } p_{2}^{*}$ is observed. This suggests a reduction of flow separation and corresponds to the findings of King & Smith on the oscillatory flow in a diffuser [11].

**Fig. 5:**
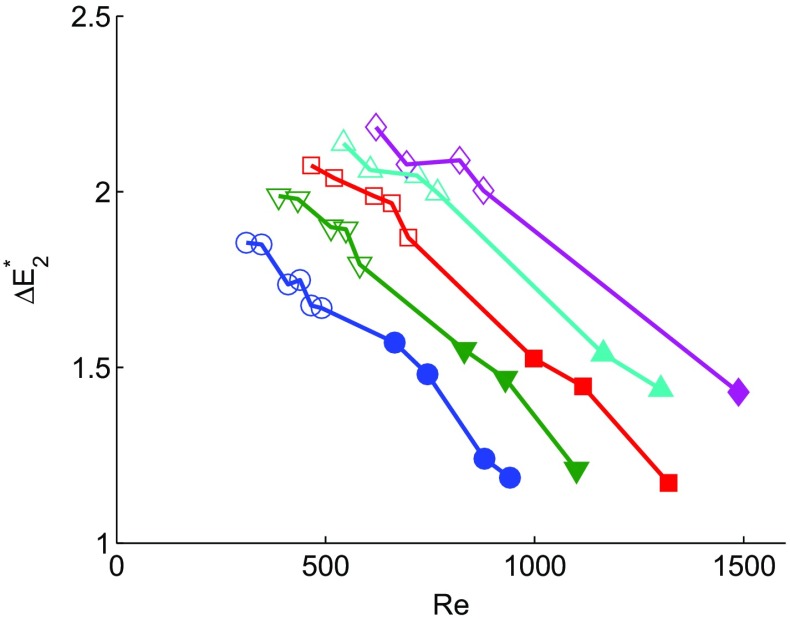
Dimensionless acoustic power dissipation measured from two jet pump samples as a function of the acoustic Reynolds number. Each curve represents results at a fixed Keulegan-Carpenter number: *K* *C*_*α*_= 1.0 (●), 1.25 ($\blacktriangledown $), 1.5 ($\blacksquare $), 1.75 ($\blacktriangle $) and 2.0 ($\blacklozenge $). The different measurement points are obtained by varying the jet pump geometry (closed symbols for *α*=7^∘^, open symbols for *α*=15^∘^) and driving frequency from 40 Hz to 100 Hz

The effect of flow separation on the jet pump performance is further emphasized by studying the dimensionless acoustic power dissipation (5). By using two different taper angles and varying the driving frequency, the ratio between *K* *C*_*α*_ and *R* *e* is influenced. Hence, the relation between the tendency to flow separation (*K* *C*_*α*_) and the momentum of the fluid (*R* *e*) can be studied. Figure 5 shows the dimensionless acoustic power dissipation as a function of the Reynolds number for various values of *K* *C*_*α*_ in the regime where flow separation can occur. For a given value of *K* *C*_*α*_, the dimensionless acoustic power dissipation decreases with the Reynolds number. Hence, an increase in Reynolds number leads to a reduction of the energy dissipated in the jet pump, which can only be understood if less energy dissipating flow features, such as flow separation, are present. A higher Reynolds number results in more boundary layer energy to withstand the adverse pressure gradient that ultimately causes the flow to separate [11]. The described observations will be further discussed in the next section, supported by velocity measurements close to the jet pump.

## Flow Separation and Vortex Propagation

**Fig. 6:**
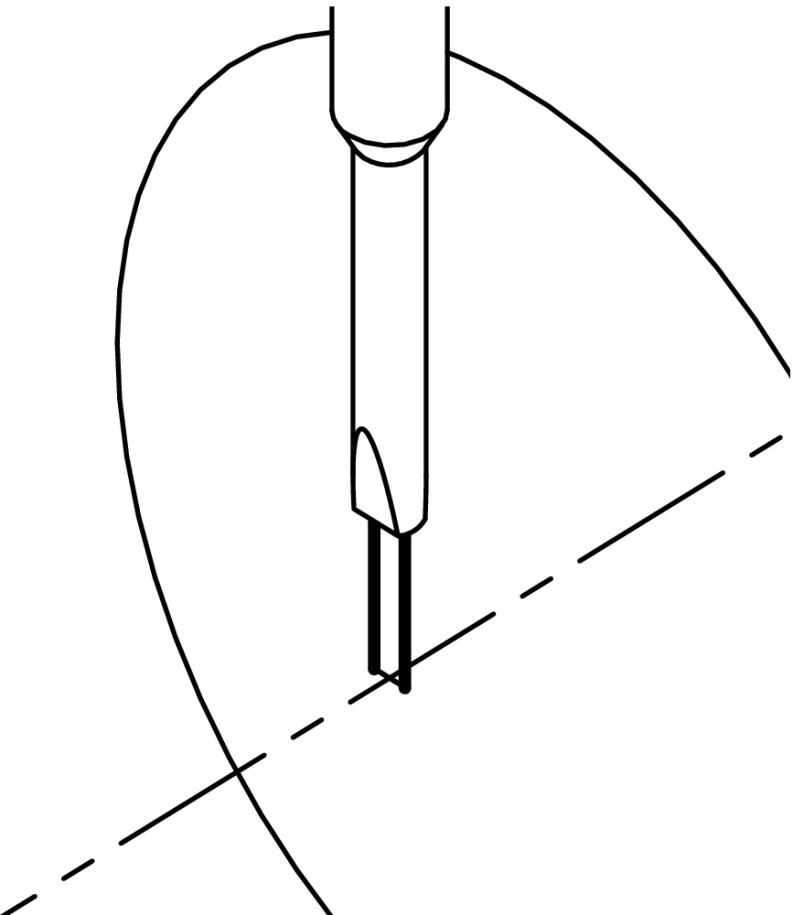
Orientation of the Dantec hot-wire probe mounted just outside the big opening of the jet pump (isometric view). The actual hot-wire is situated between the two prongs. The dashed line indicates the jet pump centerline

The onset of flow separation coincides with vortices propagating leftward from the jet pump waist through the jet pump during one half of the acoustic period [9]. As such, the occurrence of flow separation can be identified by capturing the leftward vortex propagation. The latter is performed by using hot-wire anemometry to measure the local velocity just outside the jet pump’s big opening. A single hot-wire probe is mounted at the centerline, an axial distance of 5 mm from the jet pump (indicated by “HW” in Fig. 3 and shown in detail in Fig. 6). The probe is oriented such that the plane spanned by the wire and the wire-prong is perpendicular to the wave propagation direction to minimize the intrusiveness of the hot-wire probe on the flow. A calibration is performed under the same hot-wire orientation using a calibration nozzle in steady flow [17]. Velocities between 1.8 m/s to 40 m/s have been calibrated against the pressure drop over the calibration nozzle, which is measured using a water column with a resolution of 1 Pa. This yields an uncertainty in the velocity of less than 5 % for velocities higher than 4 m/s. The accuracy of the hot-wire measurements is verified by comparing the velocity amplitude with the calculated jet pump waist velocity amplitude (see Section 2). Assuming incompressible expansion through the jet pump, the velocity amplitude at the hot-wire location is estimated. For operating conditions where no flow separation is expected (*K* *C*_*α*_<0.7), the difference is less than 0.5 m/s. It must be noted that the static calibration method is suitable for velocity amplitude measurements, while errors might occur in measuring velocities around flow reversal [18, 19]. This is considered acceptable for the current purpose of hot-wire measurements. The platinum coated tungsten hot-wire has a diameter of 5 μm, a length of 0.73 mm and is used in combination with a Dantec 90C10 Constant Temperature Anemometer (CTA) module [17]. The bandwidth is 75 kHz as was determined using the internal square wave test of the CTA module. The hot-wire signal is captured on a separate system using a NI-9215A BNC data acquisition system at a sampling frequency of *f*_*s*_=20 kHz and a sampling time of *T*_*s*_=60 s for each setpoint. Note that with this single hot-wire configuration no distinction can be made between flow in the left and right directions. This means that a pure, harmonic velocity oscillation will lead to a signal shape corresponding to a rectified sine wave and, consequently, to a peak in the frequency spectrum at twice the driving frequency. Any streaming occurring might lead to a shift in the velocity signal, resulting in an altered signal shape. The method used to identify the flow separation regardless of the velocity signal shape is described in Section 4.2.

**Fig. 7:**
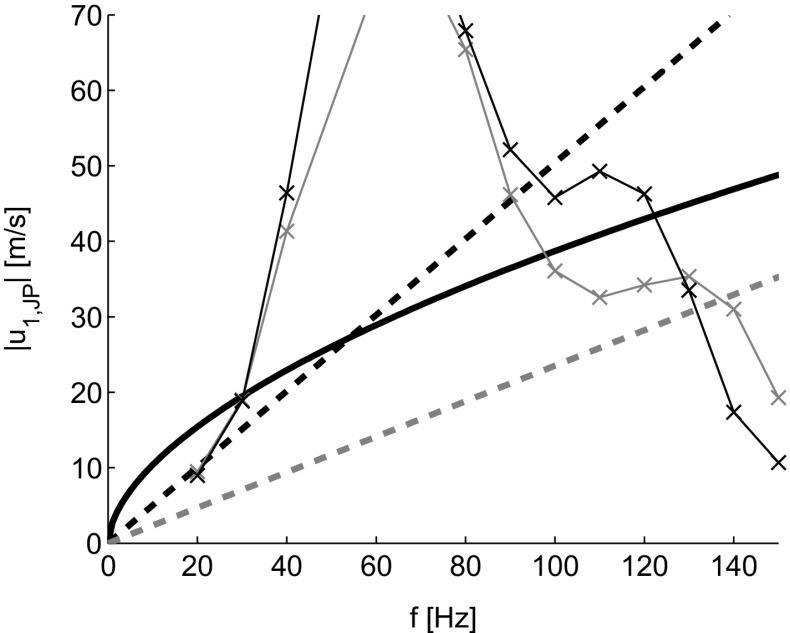
Theoretical boundaries of flow regimes as a function of the driving frequency and jet pump velocity amplitude. Black solid line indicates *R* *e* = *R* *e*_*c*_, dashed lines indicate *K* *C*_*α*_=0.7 for *α*=7^∘^ jet pump (*black*) and *α*=15^∘^ jet pump (*gray*). Thin lines indicate jet pump velocity amplitude at maximal audio signal amplification attainable with the experimental setup using *α*=7^∘^ (*black*) and *α*=15^∘^ (*gray*) jet pump

Measurements are carried out with the two jet pump samples described in Section 3 at three different driving frequencies, *f*= 40 Hz, 80 Hz, and 100 Hz, over the full range of wave amplitudes achievable with the experimental setup. The driving frequency and jet pump geometry have an influence on the velocity amplitude where either flow separation (*K* *C*_*α*_>0.7) or turbulence (*R* *e*>*R* *e*_*c*_) can be expected. Figure 7 shows the theoretical boundary between laminar and turbulent flow (thick solid line). The onset of flow separation is shown by the dashed lines. For the 15 ^∘^ jet pump (lower gray line) the onset of flow separation is expected at lower amplitudes than the transition to turbulence for the investigated frequency range. The two lines cross for the 7 ^∘^ jet pump at a frequency of 54 Hz. Given the maximum achievable jet pump velocity amplitude with the current experimental setup as a function of the driving frequency (thin lines), the turbulent regime can be reached when driving the setup at a frequency between 40 Hz to 100 Hz. Hence, this frequency range is chosen for the characterization of the onset of both flow separation and turbulence in the two different jet pump samples.

### Velocity time traces

**Fig. 8:**
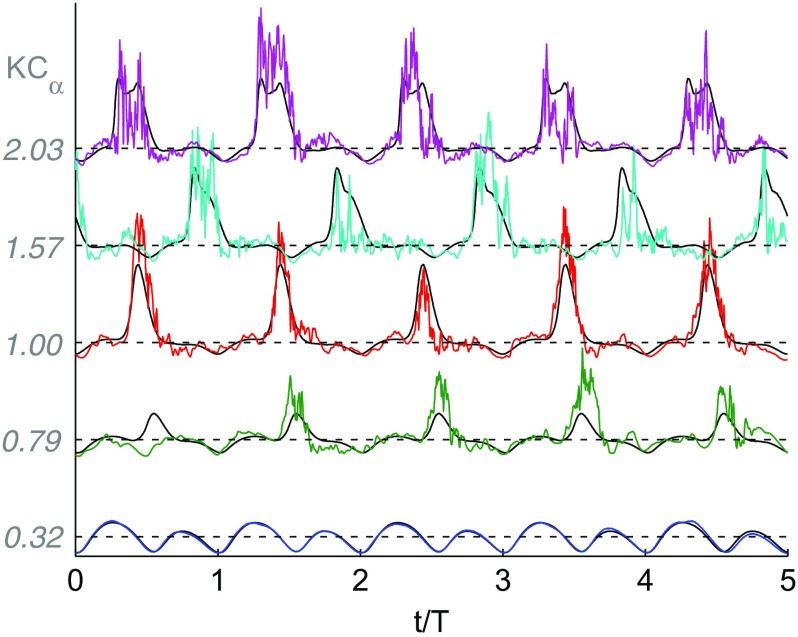
Velocity recordings during five consecutive wave periods for *α*=15^∘^ jet pump, *f*=40 Hz. Traces shown at *K* *C*_*α*_= 0.32, 0.79, 1.00, 1.57 and 2.03 corresponding to *R* *e*= 98, 243, 311, 487 and 632, respectively. Lines are vertically displaced and normalized by the median of the phase-averaged velocity (9) to enhance readability. Black solid lines represent phase-averaged velocity, five times repeated in time

A sweep over wave amplitude, equal to the jet pump performance measurements carried out in Section 3, is executed for each jet pump sample and driving frequency. The velocity signal is recorded for 60 s per setpoint. This results in 2400 to 6000 wave periods captured per setpoint, depending on the frequency. Figure 8 shows the velocity signal for five consecutive periods at various values of *K* *C*_*α*_ for the 15 ^∘^ taper angle jet pump driven at 40 Hz. From all the recorded wave periods, a phase-averaged velocity is calculated

8 $$ \left\langle{u}\right\rangle(\tau) = \frac{1}{N_{p}}\sum\limits_{i=1}^{N_{p}}{u\left( \tau+(i-1)\cdot T\right)},  $$

with *T*=1/*f* the wave period, *τ* a relative time ranging from 0 to *T* and *N*_*p*_ the total number of wave periods recorded. The phase-averaged velocity, which is still a function of the relative time *τ* or equivalently the wave phasing *φ*, is shown by the overlaying black solid lines in Fig. 8. The bottom velocity trace at *K* *C*_*α*_=0.32 shows a clean acoustic profile. Hardly any high-frequency perturbations are observed, which is reasonable given that *R* *e*≪*R* *e*_*c*_. The signal has a typical rectified sine shape due to the directional ambiguity of the velocity derived from the hot-wire signal. When the wave amplitude is increased to *K* *C*_*α*_=0.79, a periodic burst in the velocity is observed. This periodic burst is also visible at higher wave amplitudes (*K* *C*_*α*_= 1.0, 1.57 and 2.03 in Fig. 8). Furthermore, the amount of high-frequency perturbations increases when the Reynolds number increases. In Section 5 these effects will be further quantified.

### Identification of flow separation and vortex propagation

To identify the onset of flow separation and the related leftward vortex propagation, the recorded phase-averaged velocity profile is examined. When a vortex passes the hot-wire probe, a periodic burst in the signal is expected due to the temporal high absolute velocity. This is confirmed from CFD simulations where a periodic peak in the velocity signal just outside the jet pump is observed when flow separation occurs [9].

**Fig. 9:**
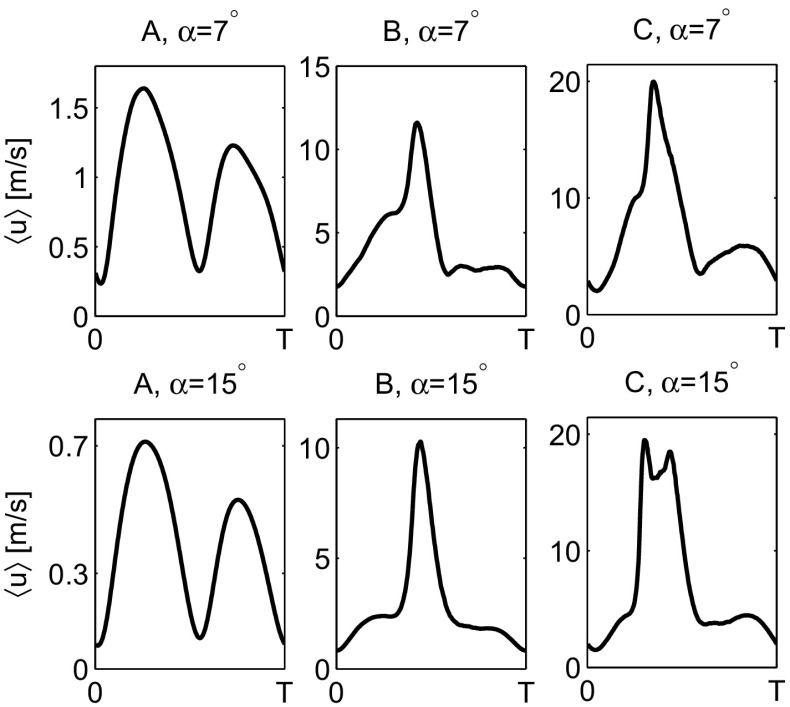
Typical shapes of the phase-averaged velocity $\left \langle {u}\right \rangle $ with *K* *C*_*α*_ increasing from A to C: pure acoustic profile, *K* *C*_*α*_≈0.3 (A); flow separation, single peak, *K* *C*_*α*_≈1.0 (B) and *K* *C*_*α*_≈2.0 (C). Results shown for the *α*=7^∘^ jet pump (top row) and the *α*=15^∘^ jet pump (bottom row), *f*=40 Hz

From the hot-wire measurements, three general shapes of the phase-averaged velocity profile are distinguished and shown in Fig. 9 A–C for the 7 ^∘^ taper angle jet pump (top row) and the 15 ^∘^ taper angle jet pump (bottom row) at *f*=40 Hz. At low amplitudes (*K* *C*_*α*_≪0.7, Fig. 9A), 〈*u*〉 has a harmonic shape at twice the driving frequency due to the directional ambiguity of the hot-wire signal. When the amplitude is increased, a separate peak starts to appear in the velocity profiles (Fig. 9B) and the velocity profile shows exactly the same features observed in numerical results when leftward vortex shedding and flow separation occurs. At even higher amplitudes (Fig. 9C, *α*=15^∘^) two close sharp peaks are measured. In numerical results this additional peak is also observed and linked to an interaction of the emerging vortex with weak vortex rings that are generated from the edge of the large jet pump opening [9]. For cases with similar *K* *C*_*α*_ but a larger Reynolds number (i.e., at higher frequencies or lower jet pump taper angles than 40 Hz and 15 ^∘^) the secondary peak is less prominent and probably dimmed by a larger turbulent intensity. This is visible in the top right plot of Fig. 9 for the *α*=7^∘^ jet pump.

The height of the periodic peak in the phase-averaged velocity profile caused by leftward vortex propagation, will be used to identify the occurrence of flow separation. To calculate the height of this peak, first an appropriate baseline value from the phase-averaged velocity profile is defined to avoid any mean velocity from affecting the calculated peak. To avoid the flow separation peak itself from influencing this baseline velocity, the median is used instead of the arithmetic mean,

9 $$ \overline{\left\langle{u}\right\rangle} = \text{median}\left( \left\langle{u}\right\rangle\right).  $$

Then, the peak height is defined as the distance between the maximum and baseline value of the phase-averaged velocity profile,

10 $$ u_{pk} = \max{\left\langle{u}\right\rangle}-\overline{\left\langle{u}\right\rangle}.  $$

**Fig. 10:**
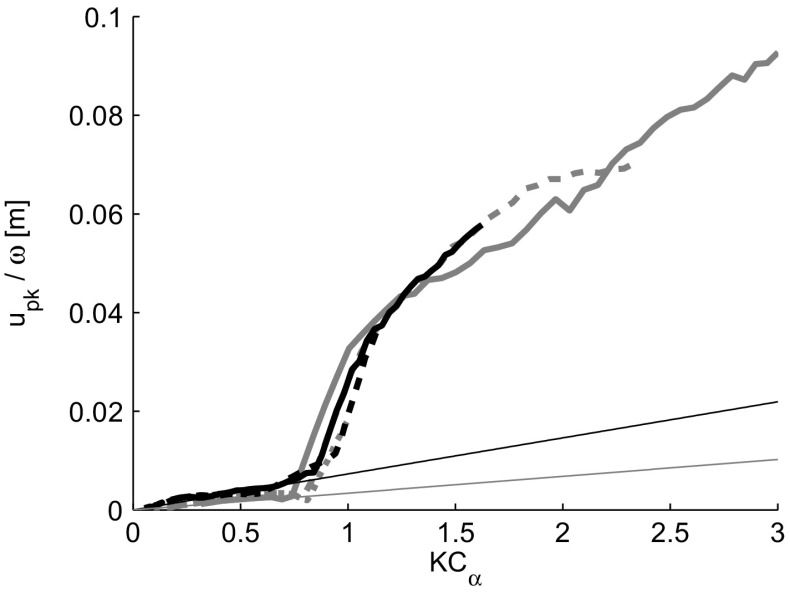
Peak height *u*_*pk*_ in phase-averaged velocity profile (10) scaled by the angular frequency *ω* and shown as a function of *K* *C*_*α*_. Black lines indicate *α*=7^∘^ jet pump, gray lines indicate *α*=15^∘^ jet pump. Line styles represent different frequencies: 40 Hz (solid), 80 Hz (dashed) and 100 Hz (dotted). The thin lines show the theoretical course of *u*_*pk*_/*ω* in the case of a purely sinusoidal velocity without flow separation

Figure 10 shows the calculated velocity peak as a function of *K* *C*_*α*_ for both jet pump samples and all driving frequencies. By dividing *u*_*pk*_ by the (angular) frequency, the contribution of the frequency to the magnitude of the velocity peak is correctly accounted for and all cases collapse to a single curve with *u*_*pk*_/*ω* representing an instantaneous displacement amplitude. The thin lines in Fig. 10 indicate the theoretical course of *u*_*pk*_/*ω* if the flow would be purely oscillatory, i.e. when no flow separation or vortex shedding occurs. This corresponds to a pure sinusoid, assuming the volume flow rate to be equal at the hot-wire location and in the jet pump waist. The theoretical course of *u*_*pk*_/*ω* is well approached by the measured values up to the point where the flow separation and leftward vortex propagation is initiated. In all cases, a clear increase in the peak is observed around *K* *C*_*α*_=0.7, which matches well with the onset of flow separation determined in a previous numerical study [10]. It becomes clear that the Keulegan-Carpenter number is indeed the parameter that determines the onset of flow separation and that the effect of the jet pump taper angle is nicely accounted for in *K* *C*_*α*_.

Using the magnitude of the velocity peak as a measure for the vorticity of the leftward propagating vortex, it can be concluded that the Reynolds number has no effect on the strength of the vortex generated. For a given *K* *C*_*α*_, the Reynolds number differs approximately by a factor of two between the 7 ^∘^ and 15 ^∘^ jet pump samples. As the curves in Fig. 10 overlay, it becomes clear that there is no effect of the Reynolds number on the height of the velocity peak. Alternatively, the propagation speed of the vortex is quantified by calculating the width of the velocity peak as the time that the phase-averaged velocity 〈*u*〉 exceeds its median value $\overline {\left \langle {u}\right \rangle }$ incremented by the standard deviation. For all cases investigated, the peak duration converges to Δ*t*_*pk*_/*T*=0.3 for *K* *C*_*α*_>0.7. Hence, no significant influence of the Reynolds number is observed, which is also widely reported in vortex ring literature [20–22].

This behavior might seem to be in contradiction with the measured jet pump performance introduced in Section 3 where ${\Delta } p_{2}^{*}$ showed a stabilizing tendency for *R* *e*>*R* *e*_*c*_ and the acoustic power dissipation decreased as a function of the Reynolds number for a given value of *K* *C*_*α*_. However, it is important to realize that the peak in the velocity profile is caused by the leftward vortex propagation from the jet pump waist and not directly by the flow separation itself. Although the onset of these two flow phenomena do coincide, it has been discussed that only the flow separation significantly influences the occurring minor losses [9, 11]. A detailed investigation of the flow field inside the jet pump is required to directly reveal the behavior of the flow separation as a function of the Reynolds number.

## Turbulence

Besides the leftward vortex propagation, the recorded hot-wire signals allow us to analyze the amount of turbulence generated by the jet pump. Turbulence in oscillatory pipe flow has been studied extensively [11, 16, 23]. In general, turbulence can be characterized by the acoustic Reynolds number (6) if the tube diameter is sufficiently large (*R*/*δ*_*ν*_>10). However, the Reynolds number for the oscillatory flow through jet pumps is not uniquely defined because the velocity amplitude is not constant throughout the jet pump. So far we have assumed the jet pump waist, where the velocity amplitude is maximal, to be the point where turbulence is first generated. Depending on the displacement amplitude and the amount of turbulent mixing, the generated turbulent eddies will propagate to the hot-wire measurement location where they will be registered as velocity fluctuations. After calculating the periodic contribution to the velocity signal using the phase-averaged velocity (8), the fluctuating part of the velocity is calculated from

11 $$ u^{\prime}(t) = u(t) - \left\langle{u}\right\rangle, $$

and in a similar way as the phase-averaged velocity 〈*u*〉, the standard deviation as a function of the relative time *τ* is calculated using,

12 $$ \left\langle{u^{2}}\right\rangle^{1/2}(\tau) = \sqrt{\frac{1}{N_{p}}\sum\limits_{i=1}^{N_{p}}{\left\lbrace u\left( \tau+(i-1)\cdot T\right)-\left\langle{u}\right\rangle(\tau)\right\rbrace^{2}}}.  $$

There are two phenomena affecting the phase-averaged standard deviation. First, 〈*u* ^2^〉^1/2^ increases as soon as the flow undergoes a transition to turbulence due to its random nature [24]. The second phenomenon resulting in an increased phase-averaged standard deviation is the occurrence of leftward vortex propagation. It was already visible in the velocity traces in Fig. 8 that the peaks in the velocity signal, which have been linked to the existence of leftward vortex propagation, do not always occur exactly at the same phase and vary in strength from period to period. This also results in a strong increase in the phase-averaged standard deviation. Consequently, the phase-averaged standard deviation by itself is not sufficient to uniquely identify the onset of turbulence in the current situation.

**Fig. 11:**
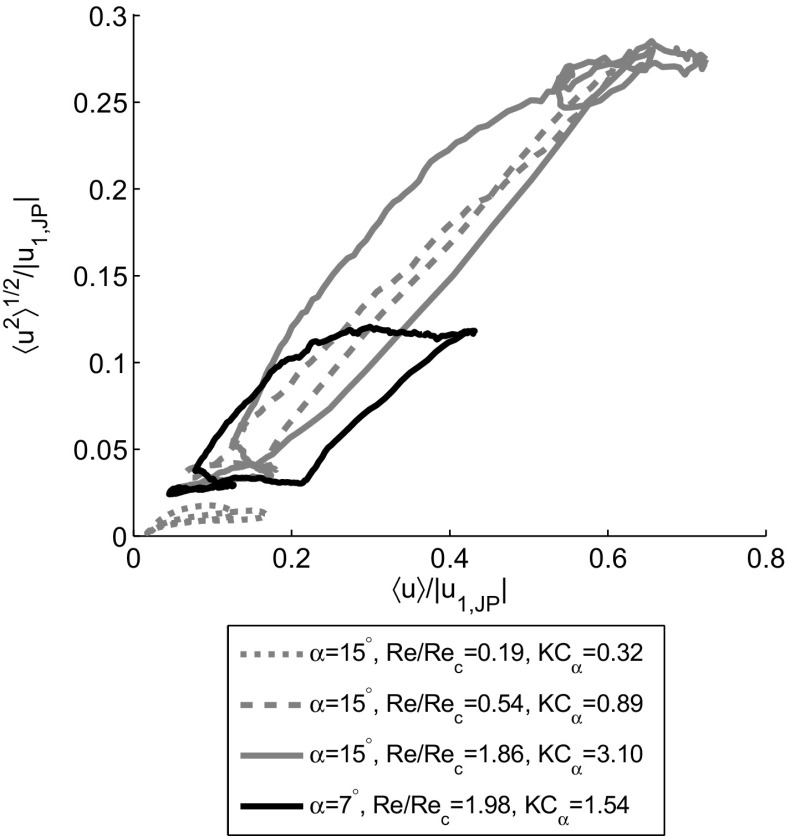
Phase-averaged standard deviation $\left \langle {u^{2}}\right \rangle ^{1/2}$ plotted against phase-averaged velocity $\left \langle {u}\right \rangle $, both normalized by the velocity amplitude in the jet pump waist |*u*_*1*,*JP*_|. Four different operating conditions shown as indicated in the legend. In all cases *f*=40 Hz

Nevertheless, there is one major difference in how the leftward vortex propagation and turbulence influence the phase-averaged standard deviation. This is illustrated in Fig. 11 by plotting the phase-averaged standard deviation against the phase-averaged velocity, both normalized using the jet pump waist velocity amplitude, and shown for various operating conditions. When only vortex propagation occurs (dashed gray line, *R* *e*/*R* *e*_*c*_=0.54 and *K* *C*_*α*_=0.89), the peak in the phase-averaged velocity occurs simultaneously with an increased phase-averaged standard deviation. This leads to a very narrow loop where the increase and decrease in both quantities follow almost the same line. When the critical Reynolds number is exceeded (black solid line, *R* *e*/*R* *e*_*c*_=1.98, *K* *C*_*α*_=1.54), the loop has a totally different shape. Following the black line in a counter-clockwise direction, the standard deviation initially stays low until a certain transition velocity is reached, then 〈*u* ^2^〉^1/2^ rapidly increases until 〈*u*〉 reaches a maximum. When the phase-averaged velocity subsequently goes down, the standard deviation does not decrease immediately but lags with respect to the phase-averaged velocity. This is caused by the relaminarization of the fluid which takes more time than the earlier transition to turbulence and occurs every period [25–27]. The wide hysteresis loops are observed for all cases where *R* *e*>*R* *e*_*c*_. The solid gray line in Fig. 11 represents a situation where both strong vortex propagation and turbulence occur for the *α*=15^∘^ jet pump. This results in a wider shape compared to the laminar case (dashed gray line). For the situation where both *R* *e* and *K* *C*_*α*_ are below their critical values (gray dotted line), no significant standard deviation is measured and due to the absence of flow separation the phase-averaged velocity stays low, even when normalized by the jet pump waist velocity amplitude. The cases shown are exemplary for all measured operating conditions, taking into account the flow regime boundaries defined by *R* *e* and *K* *C*_*α*_.

**Fig. 12:**
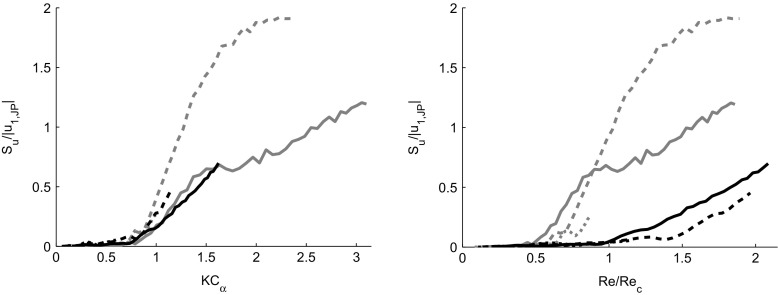
Area *S*_*u*_ enclosed by $\left (\left \langle {u}\right \rangle ,\left \langle {u^{2}}\right \rangle ^{1/2}\right )$–loops (see Fig. 11), scaled with the jet pump waist velocity |*u*_*1*,*JP*_| and plotted against *K* *C*_*α*_ (*left*) and *R* *e*/*R* *e*_*c*_ (*right*). Black lines indicate results using the *α*=7^∘^ jet pump, gray lines indicate *α*=15^∘^ jet pump. Line styles represent different frequencies: 40 Hz (*solid*), 80 Hz (*dashed*) and 100 Hz (*dotted*)

To further quantify the effect that both vortex propagation and turbulence have on the phase-averaged standard deviation, the area enclosed by the loops in Fig. 11 is calculated. Figure 12 shows the enclosed area, *S*_*u*_, for both jet pump samples (black and gray lines) and all frequencies (dotted, dashed and solid lines) as a function of *K* *C*_*α*_ (left) and *R* *e*/*R* *e*_*c*_ (right). As soon as leftward vortex propagation and flow separation occur (from *K* *C*_*α*_=0.7), resulting in narrow-shaped loops in Fig. 11, a coherent increase in the enclosed area is observed. The effect of the turbulence on *S*_*u*_ becomes visible in the right plot of Fig. 12. For *R* *e*/*R* *e*_*c*_>1, the enclosed area eventually increases with roughly the same slope for all cases. This suggests that the enclosed area is proportional to the increase in Reynolds number due to the aforementioned hysteresis in 〈*u* ^2^〉^1/2^. The fact that the different curves do not overlay one another and that the 15 ^∘^ jet pump at 40 Hz (dashed gray line) has not reached a linear increase as a function of *R* *e*/*R* *e*_*c*_ may be caused by the difference in *K* *C*_*α*_ among the various cases and thus, a different influence of the leftward vortex propagation.

**Fig. 13:**
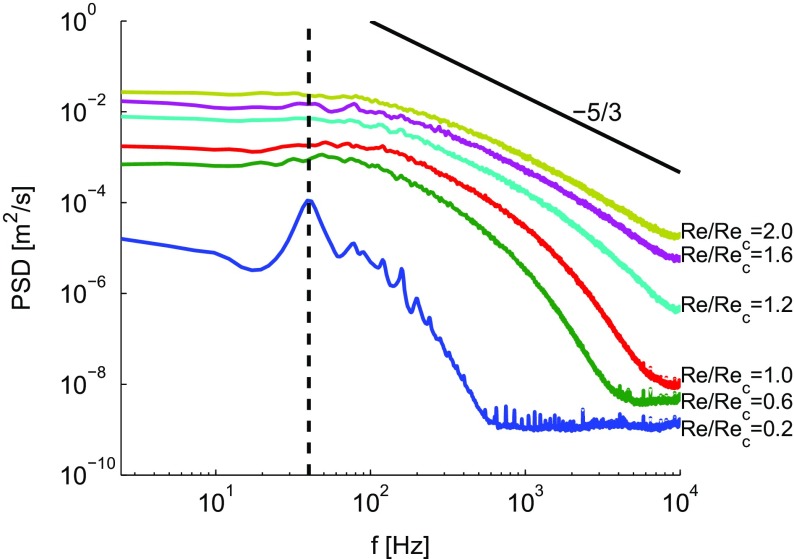
Power spectral density at various acoustic Reynolds numbers: *R* *e*/*R* *e*_*c*_= 0.2, 0.6, 1.0, 1.2, 1.6 and 2.0, using the *α*=7^∘^ jet pump driven at *f*=40 Hz as indicated by vertical dashed line. Slope of Kolmogorov turbulent spectrum is illustrated by black solid line

The applicability of the critical Reynolds number (7) as a predictor for turbulence in the oscillatory flow in jet pumps is emphasized by investigating the frequency spectra. Figure 13 shows the power spectral density (PSD) using the 7 ^∘^ jet pump driven at 40 Hz. The PSD is calculated from the fluctuating part of the velocity signal *u* ^′^, which results in a frequency spectrum where the driving frequency and all its higher harmonics are not included. The method of Welch is used to calculate the PSD where the velocity signal is divided in blocks of ten wave periods with 50 ^∘^ overlap each [28]. After applying a Hamming window, the PSD is calculated by Fourier transforming the signal. The individual lines in Fig. 13 each represent a different Reynolds number, shown as a ratio to the critical Reynolds number. The spectra for low Reynolds numbers (*R* *e*/*R* *e*_*c*_<1) decay more rapidly at higher frequencies than the higher Reynolds number spectra. As the Reynolds number exceeds its critical value, the spectra start following a −5/3 power law decay. Although the flow at the hot-wire location is far from uniform, the energy spectrum does correspond to a theoretical Kolmogorov spectrum for homogeneous isotropic turbulence [24]. For the other cases investigated, the frequency spectra show a similar behavior as a function of *R* *e*/*R* *e*_*c*_. The previous analysis underlines the applicability of a critical Reynolds number for oscillatory pipe flows (7) to jet pumps.

## Conclusion

The performance of two jet pump samples is determined experimentally in terms of the time-averaged pressure drop and the acoustic power dissipation. The results are compared against published numerical results. Good correspondence in jet pump performance is found between numerical and experimental results for Reynolds numbers in the laminar regime. However, in the experimental results the dimensionless time-averaged pressure drop stabilizes for Reynolds numbers larger than the critical Reynolds number. Furthermore, for a given Keulegan-Carpenter number *K* *C*_*α*_, the dimensionless acoustic power dissipation decreases as a function of the Reynolds number. Both of these findings indicate that the negative effect flow separation has on the jet pump performance is reduced in the turbulent regime.

Hot-wire anemometry near the jet pump big opening is used to study the onset of flow separation and turbulence in the jet pump. The occurrence of vortex propagation through the jet pump and the related flow separation is identified from periodic peaks in the recorded velocity signal. The onset of flow separation is observed from *K* *C*_*α*_>0.7 for all jet pump samples and frequencies investigated. This is fully in line with published numerical results.

Furthermore, we have shown that the Reynolds number calculated at the jet pump waist is a correct predictor for turbulence in the oscillatory flow in jet pumps. For *R* *e*>*R* *e*_*c*_ the power spectral density follows the classical −5/3 Kolmogorov spectrum. Additionally, for *R* *e*>*R* *e*_*c*_ a hysteresis in the phase-averaged standard deviation was found which is attributed to the periodic relaminarization of the fluid taking more time than the transition to turbulence.

Although the measured jet pump performance together with the defined onset of flow separation and the transition to turbulence all strongly support the hypothesis that the flow separation is reduced at high Reynolds numbers, further research is required to decisively conclude this. Supported by literature on flow separation in steady flows, the pressure gradient along the jet pump wall is of interest to determine both the location and duration of the flow separation. Moreover, the effect of the wall roughness on both the flow separation as well as on the generation of turbulence is subject to future research.

A better understanding of the flow separation inside jet pumps is shown to be key in understanding and predicting the performance of jet pumps. Design adjustments that reduce the flow separation in jet pumps with high taper angles could improve the jet pump effectiveness while maintaining a compact design.
